# ETHNIC DISPARITY IN NUTRITIONAL STATUS: EVIDENCE FROM ECUADOR’S NATIONAL SURVEY OF HEALTH, WELFARE AND AGING (SABE)

**DOI:** 10.1093/geroni/igz038.927

**Published:** 2019-11-08

**Authors:** Takashi Amano, Carlos Andres Gallegos

**Affiliations:** Brown School, Washington University in St. Louis, St. Louis, Missouri, United States

## Abstract

Providing effective public services to improve the nutritional status among older adults is pivotal in countries experiencing population aging. Public investment and social policy in Ecuador have focused mainly on standard food-based interventions and cash transference programs. These efforts, however, may be not effective enough to reach those populations that need the most. This study aims to identify those populations that should be targeted by specific interventions. Data were drawn from Ecuador’s Survey of Health, Welfare and Aging (SABE) – 2009, a probability sample of households with at least one person who were 60 years or older in Ecuador. The final sample consisted of 5,235 people who were 60 years or older. Ethnic identity was categorized into four categories: Indigenous People, Mestizo (Mixed of Spanish and Indigenous People), Afro-Ecuadorian/Mulato, and Other. Nutritional status was measured using Mini Nutritional Assessment (MNA). Ordered logistic regression analysis was utilized to assess the association between ethnic identity and nutritional status. Results revealed that the Indigenous ethnicity was significantly associated with worse nutritional status compared to Mestizo and Other even after controlling for a range of covariates including socio-economic status, health related factors, and social support. These findings suggest the existence of underlaying factors hindering the nutritional status of among indigenous older adults in Ecuador. Considering the information revealed by SABE, interventions and other strategies should be targeted and designed specifically accounting for the needs, preferences, and culture of the most vulnerable population.

